# Effect of diabetes mellitus on the development of left ventricular contractile dysfunction in women with heart failure and preserved ejection fraction

**DOI:** 10.1186/s12933-021-01379-3

**Published:** 2021-09-14

**Authors:** Ke Shi, Meng-Xi Yang, Shan Huang, Wei-Feng Yan, Wen-Lei Qian, Yuan Li, Ying-Kun Guo, Zhi-Gang Yang

**Affiliations:** 1 Department of Radiology, West China Hospital, Sichuan University, Chengdu, Sichuan China; 2 Department of Radiology, Sichuan Cancer Hospital and Institute, Sichuan Cancer Center, School of Medicine, University of Electronic Science and Technology of China, Chengdu, Sichuan China; 3 Department of Radiology, West China Second University Hospital, Sichuan University, Chengdu, Sichuan China

**Keywords:** Heart failure with preserved ejection fraction, Diabetes mellitus, Contractile dysfunction, Sex

## Abstract

**Background:**

Heart failure with preserved ejection fraction (HFpEF) is a heterogeneous syndrome with sex-specific pathophysiology. Estrogen deficiency is believed to be responsible for the development of HFpEF in women. However, estrogen deficiency does not seem to be completely responsible for the differences in HFpEF prevalence between sexes. While diabetes mellitus (DM) frequently coexists with HFpEF in women and is associated with worse outcomes, the changes in myocardial contractility among women with HFpEF and the DM phenotype is yet unknown. Therefore, we aimed to investigate sex-related differences in left ventricular (LV) contractility dysfunction in HFpEF comorbid with DM.

**Methods:**

A total of 224 patients who underwent cardiac cine MRI were included in this study. Sex-specific differences in LV structure and function in the context of DM were determined. LV systolic strains (global longitudinal strain [GLS], circumferential strain [GCS] and radial strain [GRS]) were measured using cine MRI. The determinants of impaired myocardial strain for women and men were assessed.

**Results:**

The prevalence of DM did not differ between sexes (p > 0.05). Despite a similar LV ejection fraction, women with DM demonstrated a greater LV mass index than women without DM (p = 0.023). The prevalence of LV geometry patterns by sex did not differ in the non-DM subgroup, but there was a trend toward a more abnormal LV geometry in women with DM (p = 0.072). The magnitudes of systolic strains were similar between sexes in the non-DM group (p > 0.05). Nevertheless, in the DM subgroup, there was significant impairment in women in systolic strains compared with men (p < 0.05). In the multivariable analysis, DM was associated with impaired systolic strains in women (GLS [β = 0.26; p = 0.007], GCS [β = 0.31; p < 0.001], and GRS [β = −0.24; p = 0.016]), whereas obesity and coronary artery disease were associated with impaired systolic strains in men (p < 0.05).

**Conclusions:**

Women with DM demonstrated greater LV contractile dysfunction, which indicates that women with HFpEF comorbid with DM have a high-risk phenotype of cardiac failure that may require more aggressive and personalized medical treatment.

## Background

Heart failure (HF) with preserved ejection fraction (HFpEF) is presently a global health burden, and the prevalence of HFpEF is increasing among the aging population. In contrast to HF with reduced ejection fraction, the clinical outcomes of HFpEF are still poor due to the paucity of evidence-based therapies [1]. There is a growing recognition that HFpEF is likely a heterogeneous syndrome of multiple discrete phenotypes rather than a disease entity alone [2–4].

HFpEF is the most common subtype of HF in women, and HFpEF has a higher prevalence in women than in men [5]. Studies have shown significant differences in cardiovascular remodeling between sexes, with women exhibiting left ventricular (LV) concentric remodeling, impaired diastolic function, and vascular stiffening, suggesting that a sex-specific phenotype exists in patients with HFpEF [6–8]. Mounting evidence suggests that estrogen deficiency is responsible for the development of HFpEF in women [9, 10]. However, estrogen deficiency does not seem to be completely responsible for the differences in HFpEF prevalence between sexes. As one of the most common cardiovascular risk factors, diabetes mellitus (DM) also has significant negative impacts on HFpEF development [11]. Data from registries and multicenter studies demonstrated that DM frequently coexists with HFpEF in women and is associated with worse outcomes, regardless of ejection fraction status [3, 12, 13]. Since DM could lead to ventricular hypertrophy, myocardial fibrosis, and microvascular dysfunction, it may have negative synergistic effects on elderly women that are beyond the effect of either sex or DM alone [14]. To date, limited data are available on the changes in myocardial contractility among women with HFpEF and the DM phenotype. An understanding of the sex-related differences in LV contractile function and its determinants may have important implications for the development of risk stratification tools and highlight potential phenotype-specific targets.

Accordingly, the present study aimed to compare LV contractility between women and men with HFpEF comorbid with DM using cardiac MRI strain and to investigate whether the diabetic phenotype played a distinctive role in the development of impaired myocardial contractility in women with HFpEF.

## Methods

### Study population

This study was conducted in accordance with the ethical guidelines of the 1975 Declaration of Helsinki and approved by the Biomedical Research Ethics Committees of our hospital. Written informed consent was obtained from all the patients, and the patient-sensitive data were protected with full confidentiality and used only for the purposes of this study. We enrolled patients with HFpEF between January 2018 and April 2021. HFpEF was defined according to the guidelines of the European Society of Cardiology (2019) [15], and patients who met the following criteria were enrolled in the HF group: (1) New York Heart Association class II–IV symptoms and signs of HF; (2) elevated plasma amino-terminal pro-B-type natriuretic peptide (NT-proBNP) levels (≥ 220 pg/mL for patients in sinus rhythm or ≥ 660 pg/mL for patients with atrial fibrillation); and (3) echocardiographic LV ejection fraction (LVEF) ≥ 50% accompanied by either (a) evidence of diastolic dysfunction (ratio of peak early diastolic filling velocity (E) to early diastolic mitral annular velocity (e’) ≥ 15) or (b) structural alteration of the heart, including left atrial (LA) enlargement or LV hypertrophy (LVH). Patients were excluded if they had severe valvular disease, chronic obstructive pulmonary disease, congenital heart disease, acute coronary syndrome, or pericardial disease.

### Cardiac MRI acquisition

Patients underwent MRI on a 3-T Siemens Scanner (MAGNETOM Skyra). A TurboFLASH sequence with breath-hold was performed to obtain cine images comprising a stack of contiguous short-axis slices covering the entirety of both ventricles from base to apex and one two- and four-chamber long-axis slice. The acquisition parameters were as follows: repetition time = 3.42 ms; echo time = 1.51 ms; slice thickness = 8.0 mm; flip angle = 40°; matrix = 200 × 256 pixels; and field of view = 265 × 340 mm^2^.

### Cardiac MRI postprocessing

All images were analyzed using commercially available CVI^42^ software (Circle Cardiovascular Imaging, Inc., Calgary, Alberta, Canada). We used a stack of short-axis images for LV function parameters, including LVEF, LV end-diastolic volume (LVEDV), LV end-systolic volume (LVESV), and LV stroke volume (LVSV). The results were automatically calculated after manually drawing LV endocardial and epicardial borders at the end-diastolic and end-systolic phases, respectively. LV mass (LVM) was assessed by measuring the area between the endocardial and epicardial borders in each of the short-axis slices. LV papillary muscles were counted toward the LVM but not the LV volume. LVH was defined as an LVM index > 115 g/m^2^ for men and > 95 g/m^2^ for women [16].

A stack of short-axis cine images combined with two long-axis images (one two-chamber and one four-chamber view) were loaded into the feature-tracking module for LV systolic strain analysis. During the systolic phase, LV shortens in the longitudinal and circumferential directions, causing negative global longitudinal strain (GLS) and circumferential strain (GCS), whereas thickening in the radial direction causes positive global radial strain (GRS).

The maximal LA volume (LAV) was calculated using the biplane area-length method in two- and four-chamber long-axis views at the LV end-systolic phase. LA enlargement was defined as an LAV index > 34 mL/m^2^ for patients with sinus rhythm or > 40 mL/m^2^ for patients with atrial fibrillation [15].

### Echocardiography

Echocardiographic data were obtained using the Philips echocardiographic system (IE 33 or 7500). LVEF was derived according to the modified biplane Simpson’s method. Peak early diastolic filling velocity (E) and late diastolic filling velocity during atrial contraction (A) of mitral inflow were recorded by pulsed wave Doppler. The E/A ratio was calculated. Mitral annular tissue velocity during early filling (e’) was measured by averaging the values detected at the septal and lateral annulus using tissue Doppler. E/e’ was calculated to obtain an estimate of LV filling pressure. Pulmonary artery systolic pressure (PASP) was estimated using Doppler tricuspid regurgitant velocity (V_max_) using the following equation: PASP = 4 × (V_max_)^2^ + 10 mmHg. Relative wall thickness (RWT) was calculated as twice the LV posterior wall thickness divided by the LV internal diameter at the end-diastolic phase. Four patterns of LV geometry were described: normal (RWT ≤ 0.42 and no LVH), concentric remodeling (RWT > 0.42 and no LVH), concentric hypertrophy (RWT > 0.42 and LVH), and eccentric hypertrophy (RWT ≤ 0.42 and LVH) [15].

### Statistical methods

Statistical analyses were performed using SPSS (IBM SPSS Inc., Armonk, New York, USA) and Prism (GraphPad software Inc., San Diego, California, USA) software. The normality of data was determined using the Shapiro–Wilk test. Continuous variables were presented as the means with standard deviations or medians with interquartile ranges (IQRs). Categorical variables were presented as counts and percentages. Differences in baseline characteristics and echocardiographic and cardiac MRI findings between groups were examined using Student’s t-test, the Wilcoxon–Mann–Whitney test, or the chi-square test (Fisher’s exact test), as appropriate. The determinants of impaired myocardial strain for women and men were obtained using binary logistic regression models with a backward selection procedure. Candidate variables with a p-value < 0.10 in the univariable analysis and the absence of collinearity were included in the final multivariable model. Differences with a two-tailed p-value < 0.05 were considered statistically significant.

## Results

### Baseline characteristics of the study population by sex

A total of 224 patients with HFpEF were finally included in this study. Women were on average 4.0 years older than men (p = 0.019) and had a higher body mass index (BMI) (p = 0.043), but heart rate, blood pressure, and HF duration were similar between sexes (Table 1). Compared with men, women displayed a similar prevalence of cardiovascular comorbidities, including DM, hypertension, and atrial fibrillation. However, coronary artery disease (CAD) was less common in women (women, 17.6%, vs. men, 30.2%; p = 0.028). Women had higher median plasma concentrations of NT-proBNP (p = 0.004), total triglycerides (p = 0.042), and cholesterol (p = 0.033) but a lower mean estimated glomerular filtration rate (p = 0.013). There were no significant differences in the use of medications, except for statins, between sexes (women, 26.9%, vs. men, 39.7%; p = 0.042).

**Table 1:** Baseline characteristics of the study population by sex

	Women (n = 108)	Men (n = 116)	p-value
Age, yrs	63.3 ± 9.4	59.3 ± 11.3	0.019
BMI, kg/m^2^	23.1 ± 3.9	24.2 ± 3.3	0.043
Heart rate, beats/min	83.2 ± 14.4	79.2 ± 17.1	0.118
SBP, mmHg	126.4 ± 21.2	121.7 ± 21.9	0.181
DBP, mmHg	76.1 ± 13.5	73.7 ± 14.9	0.297
PP, mmHg	50.3 ± 17.2	48.0 ± 19.3	0.445
HF duration			
≤ 1 yr	63 (58.3)	63 (54.3)	0.786
> 1 and ≤ 5 yrs	27 (25.0)	30 (25.9)	
> 5 yrs	18 (16.7)	23 (19.8)	
Cardiovascular comorbidities, n (%)			
DM	47 (43.5)	43 (37.1)	0.325
HT	67 (62.0)	75 (64.7)	0.684
CAD	19 (17.6)	35 (30.2)	0.028
Cardiovascular risk factors, n (%)			
Smoking	4 (3.7)	57 (49.1)	< 0.001
Atrial fibrillation	24 (22.2)	24 (20.7)	0.780
Dyslipidemia	34 (31.5)	26 (22.4)	0.126
Medications, n (%)			
Beta-blocker	71 (65.7)	75 (64.7)	0.865
ACEI/ARB	77 (71.3)	86 (74.1)	0.633
Diuretics	62 (57.4)	76 (65.5)	0.212
CCB	15 (13.9)	16 (13.8)	0.983
Anti-thrombotic agents	48 (44.4)	58 (50.0)	0.405
Statins	29 (26.9)	46 (39.7)	0.042
Laboratory data			
NT-proBNP, pg/mL	1970 (698–3967)	1696 (586–3994)	0.004
eGFR, mL/min/1.73m^2^	70.8 ± 26.5	76.9 ± 25.7	0.013
TG, mmol/L	1.4 (0.9–1.9)	1.2 (0.8–1.6)	0.042
TC, mmol/L	4.2 (3.4–5.1)	3.9 (3.2–4.5)	0.033
HDL-C, mmol/L	1.1 (0.9–1.4)	1.0 (0.8–1.3)	0.181

Data are presented as mean ± SD, media (Q1–Q3) or number (percentage)

BMI: body mass index; SBP: systolic blood pressure; DBP: diastolic blood pressure; PP: pulse pressure; HF: heart failure; DM: diabetes mellitus; HT: hypertension; CAD: coronary artery disease; AF: atrial fibrillation; ACEI: angiotensin converting enzyme inhibitor; ARB: angiotensin receptor blocker; CCB: calcium-channel blocker; NT-proBNP: amino-terminal pro-B-type natriuretic peptide; eGFR: estimated glomerular filtration rate; TG: triglycerides; TC: cholesterol; HDL-C: high-density lipoprotein cholesterol content

Among the overall cohort, 90 patients with HFpEF (90/224, 40.2%) had DM, of whom 47 (47/90, 52.2%) were women. No significant differences were observed in hemoglobin A1c levels (women, 6.2 [IQR, 5.5–7.8]%, vs. men, 6.6 [IQR, 6.2–7.8]%; p = 0.283) or antidiabetic treatment between sexes (data not shown, p > 0.05).

### Sex-related differences in cardiac structure and function in HFpEF comorbid with DM

**Fig. 1:**
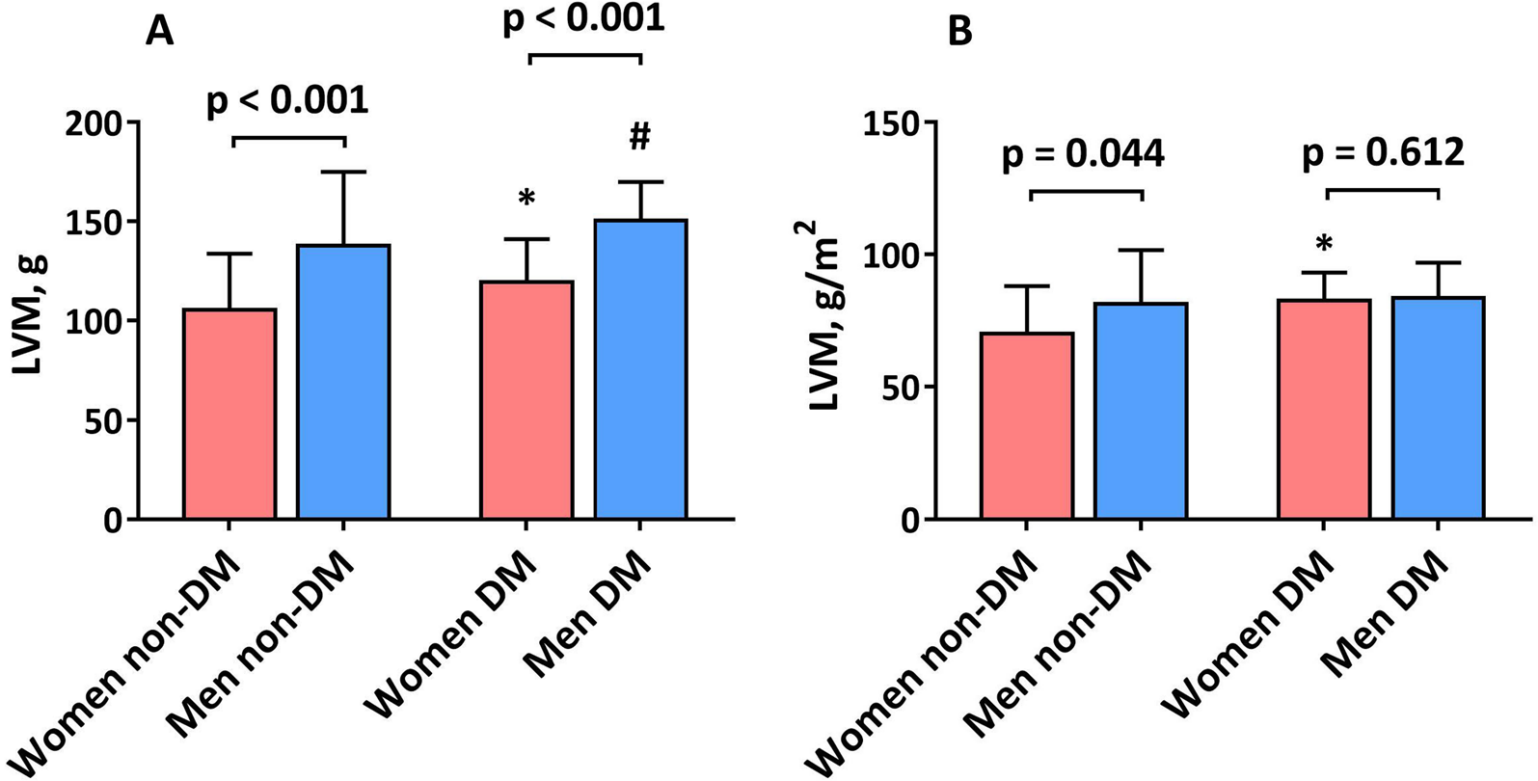
Left ventricular mass (LVM) between sexes with and without diabetes mellitus (DM). *p-value < 0.05 vs. women without DM. #p-value < 0.05 vs. men without DM

**Fig. 2:**
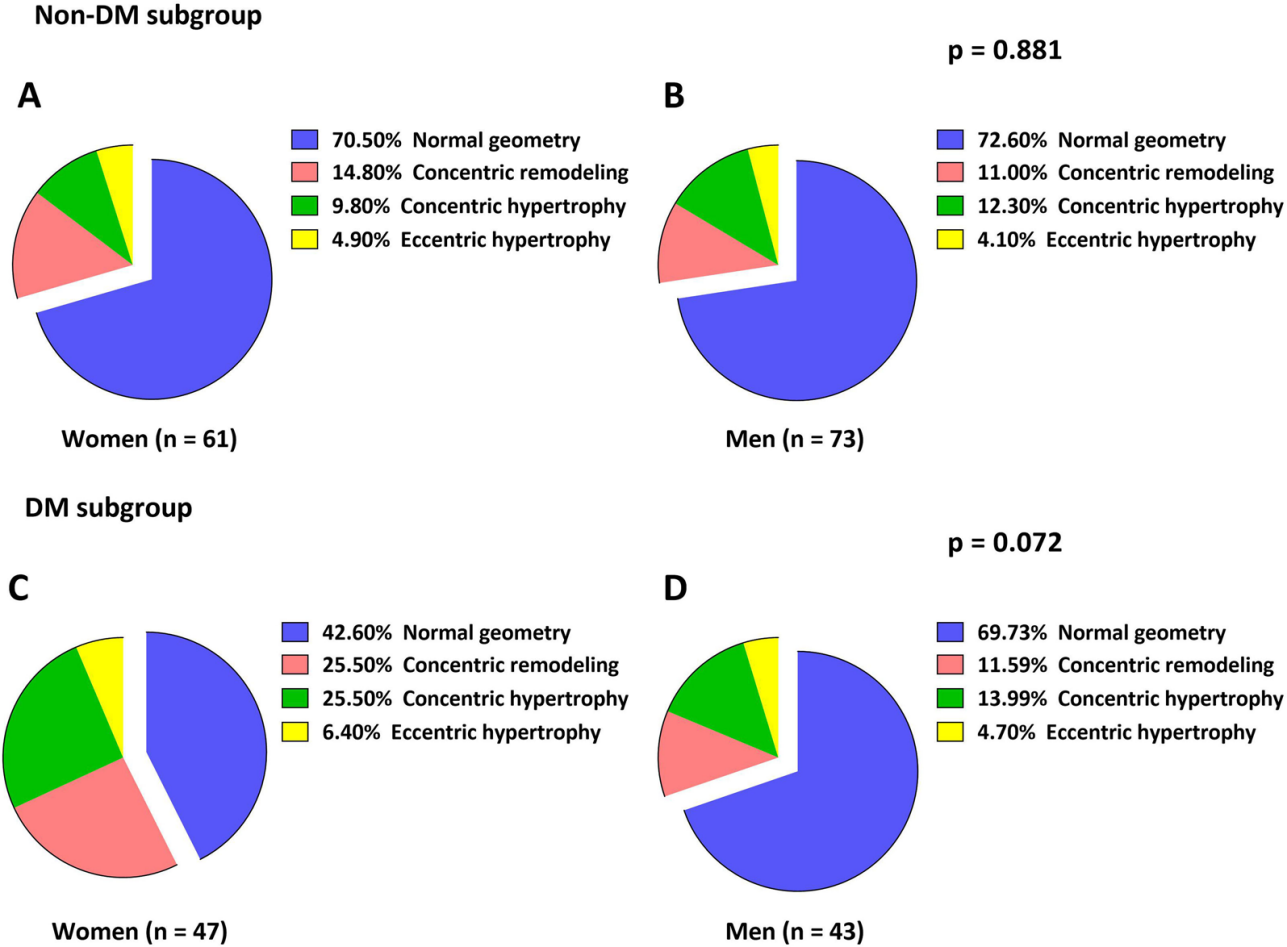
Prevalence of left ventricular geometry distribution between sexes with and without diabetes mellitus (DM)

As shown in Table 2, although LVEF was similar in men and women, LVEDV, LVESV, and LVSV were lower in women regardless of DM status (all p < 0.05). However, when corrected for body size, these indices did not notably differ between sexes (all p > 0.05). The LVM index was lower in women than in men in the non-DM subgroup (70.0 [IQR, 62.5–88.1] g/m^2^ vs. 81.3 [IQR, 67.6–101.7] g/m^2^, p = 0.044; Fig. 1A), whereas the difference between sexes in the DM subgroup was not significant (82.9 [IQR, 67.6–90.1] g/m^2^ vs. 83.5 [IQR, 70.7–96.9] g/m^2^, p = 0.612; Fig. 1B). Moreover, the prevalence of LV geometry patterns by sex did not differ in patients without DM (p = 0.881; Fig. 2A, B). In contrast, there was a trend toward more concentric remodeling and hypertrophy in women with DM than in men with DM (p = 0.072; Fig. 2C, D). Moreover, although the LAV index was similar between sexes, there was a trend toward a smaller LAV index in women with DM than in women without DM (37.9 ± 15.1 mL/m^2^ vs. 44.5 ± 22.2 mL/m^2^; p = 0.242), as was also the case in men with and without DM (37.3 ± 19.2 mL/m^2^ vs. 43.6 ± 19.8 mL/m^2^; p = 0.215).

**Table 2:** Sex-related differences of cardiac structure and function in HFpEF patients with and without DM

	Nondiabetic patients (n = 134)		p-value	Diabetic patients (n = 90)		p-value
	Women (n = 61)	Men (n = 73)		Women (n = 47)	Men (n = 43)	
Cardiac MRI parameters						
LVEDV, mL	108.9 ± 22.4	131.0 ± 14.1	< 0.001	114.8 ± 27.1	142.4 ± 39.4	0.019
LVEDV index, mL/m^2^	75.2 ± 16.3	80.4 ± 11.6	0.182	78.3 ± 18.7	81.0 ± 23.3	0.709
LVESV, mL	46.3 ± 14.4	58.8 ± 9.9	< 0.001	50.5 ± 17.9	67.3 ± 23.5	0.021
LVESV index, mL/m^2^	31.9 ± 10.5	36.2 ± 7.6	0.097	34.4 ± 11.8	38.9 ± 15.3	0.337
LVSV, mL	62.7 ± 15.0	72.2 ± 10.3	0.010	64.4 ± 19.8	75.1 ± 22.8	0.141
LVSV index, mL/m^2^	43.2 ± 10.5	44.2 ± 7.2	0.683	43.9 ± 13.9	42.1 ± 11.8	0.679
LVEF, %	58.6 ± 7.9	55.7 ± 4.9	0.123	58.9 ± 7.5	56.5 ± 6.4	0.304
LVM, g	105.8 (95.1–129.8)	137.7 (112.3–175.1)	< 0.001	119.5 (100.7–133.2)^*^	150.5 (129.7–169.9)^#^	< 0.001
LVM index, g/m^2^	70.0 (62.5–88.1)	81.3 (67.6–101.7)	0.044	82.9 (67.6–90.1)^*^	83.5 (70.7–96.9)	0.612
LAV, mL	66.2 ± 32.0	72.9 ± 33.0	0.374	56.4 ± 23.1^*^	53.3 ± 28.1^#^	0.705
LAV index, mL/m^2^	44.5 ± 22.2	43.6 ± 19.8	0.857	37.9 ± 15.1	37.3 ± 19.2	0.906
Echocardiographic parameters						
LVEF, %	61.0 ± 9.8	59.1 ± 8.1	0.319	62.6 ± 10.1	60.2 ± 8.7	0.343
E/A ratio	1.0 (0.6, 1.6)	1.3 (0.7, 1.9)	0.263	0.8 (0.7, 1.4)	1.3 (0.8, 1.5)	0.155
E/e’ average ratio	19.1 ± 3.0	17.3 ± 2.3	0.025	20.3 ± 2.9	18.1 ± 3.1	0.006
RWT	0.38 (0.35–0.42)	0.39 (0.33–0.42)	0.220	0.40 (0.36–0.43)	0.40 (0.34–0.42)	0.540
PASP, mmHg	36.9 ± 4.9	34.8 ± 8.1	0.295	36.2 ± 7.9	37.0 ± 7.9	0.747

Data are presented as mean ± SD, or media (Q1–Q3)

HFpEF: heart failure with preserved ejection fraction; DM: diabetes mellitus; LVEDV: left ventricular end-diastolic volume; LVESV: left ventricular end-systolic volume; LVSV: left ventricular stroke volume; LVEF: left ventricular ejection fraction; LVM: left ventricular mass; LAV: left atrial volume; E: peak early diastolic filling velocity; A: late diastolic filling velocity during atrial contraction; e’: mitral annular tissue velocity during early filling; RWT: relative wall thickness; PASP: pulmonary artery systolic pressure

*p-value < 0.05 vs. women without DM

^#^p-value < 0.05 vs. men without DM

The E/e’ ratio was higher in women than men in both the non-DM (19.1 ± 3.0 vs. 17.3 ± 2.3; p = 0.025) and DM subgroups (20.3 ± 2.9 vs. 18.1 ± 3.1; p = 0.006), suggesting more diastolic dysfunction in women. In addition, there was a trend toward a lower E/A ratio in women than in men in both the non-DM (p = 0.263) and DM subgroups (p = 0.155). No significant differences in RWT between sexes in either the non-DM or DM subgroups were observed (p > 0.05).

### Sex-related differences in the effect of DM on LV contractility

**Fig. 3:**
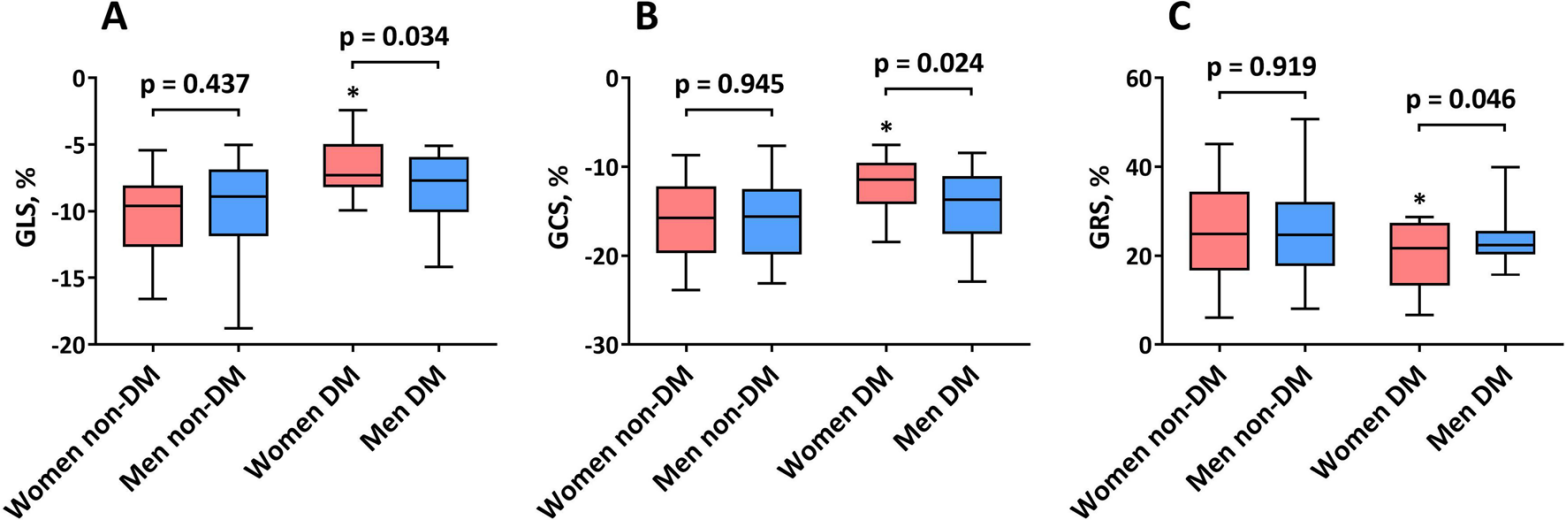
Global left ventricular systolic strain indices between sexes with and without diabetes mellitus (DM). *p-value < 0.05 vs. women without DM. GLS: global longitudinal strain; GCS: global circumferential strain; GRS: global radial strain

Figure 3 depicts strain indices derived from cardiac MRI cine images. The magnitudes of GLS, GCS, and GRS were similar between sexes in patients without DM (all p > 0.05). However, in the DM subgroup, women exhibited more severe impairment in the magnitude of GLS (women, − 6.6% ± 2.2%, vs. men, − 8.0% ± 2.4%; p = 0.034), GCS (women, − 11.9% ± 2.8%, vs. men, − 14.1% ± 3.9%; p = 0.024) and GRS (women, 19.9% ± 7.1%, vs. men, 23.7% ± 5.4%; p = 0.045) than men.

In addition, more advanced deterioration in the magnitude of GLS (DM, − 6.6% ± 2.2%, vs. non-DM, − 10.1% ± 2.8%; p < 0.001), GCS (DM, − 11.9% ± 2.8%, vs. non-DM, − 15.8% ± 4.5%; p = 0.003), and GRS (DM, 19.9% ± 7.1%, vs. non-DM, 25.4% ± 10.9%; p = 0.039) was noted in women with DM than in women without DM, whereas no significant differences were observed for these systolic strain indices in men with and without DM (all p > 0.05).

### Sex-related differences in determinants of impaired LV contractility

The univariable analysis in women showed that age, DM, NT-proBNP level, and LVM were common variables associated with impaired GLS, GCS, and GRS (all p < 0.05) (Table 3). After multivariable adjustment, DM remained the independent determinant of impaired GLS (β = 0.26; p = 0.007), GCS (β = 0.31; p < 0.001), and GRS (β = −0.24; p = 0.016) in women.

**Table 3:** Determinants of LV contractile dysfunction in women with HFpEF

	GLS				GCS				GRS			
	Univariable		Multivariable		Univariable		Multivariable		Univariable		Multivariable	
	β	*p*-value	β	*p*-value	β	*p*-value	β	*p*-value	β	*p*-value	β	*p*-value
Age^#^, yrs	0.53	< 0.001	**0.26**	**0.027**	0.41	< 0.001	**0.22**	**0.035**	− 0.31	0.005	**-0.25**	**0.041**
Obesity^&^	0.23	0.039			0.08	0.490			− 0.16	0.156		
PP^#^, mmHg	0.09	0.421			0.06	0.611			0.02	0.889		
HT	0.08	0.481			0.14	0.207			− 0.11	0.335		
DM	0.49	< 0.001	**0.26**	**0.007**	0.39	< 0.001	**0.31**	**< 0.001**	− 0.31	0.007	**− 0.24**	**0.016**
CAD	0.24	0.036			0.21	0.066			− 0.08	0.503		
NT-proBNP^*^, pg/mL	0.30	0.007	**0.21**	**0.028**	0.27	0.016	0.17	0.095	− 0.39	< 0.001	**− 0.32**	**0.002**
eGFR, mL/min/1.73m^2^	− 0.09	0.423			0.01	0.910			0.03	0.787		
LVM, g	0.41	0.001	**0.20**	**0.042**	0.35	0.007	**0.27**	**0.020**	− 0.40	0.002	**− 0.28**	**0.007**

β is adjusted regression coefficient. Variables with bold font indicate statistical significance in multivariable analysis

^#^Changes in dependent variables per 10 units increase

^&^Subjects with body mass index ≥ 25 kg/m^2^ were classified as obese group that proposed by the World Health Organization for Asian populations

*NT-proBNP is log-transformed before being included in the regression analysis

LV: left ventricular; HFpEF: heart failure with preserved ejection fraction; GLS: global longitudinal strain; GCS: global circumferential strain; GRS: global radial strain; PP: pulse pressure; HT: hypertension; DM: diabetes mellitus; CAD: coronary artery disease; NT-proBNP: amino-terminal pro-B-type natriuretic peptide; eGFR: estimated glomerular filtration rate; LVM: left ventricular mass

In men, the univariable analysis showed that age, obesity, NT-proBNP level, and LVM were common variables that were associated with impaired GLS, GCS, and GRS (all p < 0.05) (Table 4). CAD was associated with impaired GLS (p = 0.002), whereas there was only a trend toward an association between DM and impaired GLS (p = 0.060). In the multivariable analysis, age, NT-proBNP level, and LVM were significantly associated with impaired GLS, GCS, and GRS (all p < 0.05). However, CAD was independently associated only with impaired GLS (p = 0.037). Obesity was associated with impaired GCS and GRS, and showed a trend toward an association with impaired GLS (p = 0.058).

**Table 4:** Determinants of LV contractile dysfunction in men with HFpEF

	GLS				GCS				GRS			
	Univariable		Multivariable		Univariable		Multivariable		Univariable		Multivariable	
	β	*p*-value	β	*p*-value	β	*p*-value	β	*p*-value	β	*p*-value	β	*p*-value
Age^#^, yrs	0.42	< 0.001	**0.22**	**0.035**	0.26	0.018	**0.21**	**0.046**	− 0.17	0.007	**− 0.11**	**0.027**
Obesity^&^	0.25	0.020	0.18	0.058	0.29	0.006	**0.29**	**0.003**	− 0.28	0.009	**− 0.28**	**0.005**
PP^#^, mmHg	− 0.05	0.614			− 0.07	0.552			0.08	0.446		
HT	− 0.02	0.878			− 0.03	0.815			− 0.01	0.913		
DM	0.20	0.060			0.18	0.102			− 0.07	0.511		
CAD	0.33	0.002	**0.20**	**0.037**	0.17	0.111			− 0.14	0.189		
NT-proBNP^*^, pg/mL	0.40	< 0.001	**0.22**	**0.027**	0.25	0.022	**0.21**	**0.047**	− 0.25	0.021	**− 0.30**	**0.004**
eGFR, mL/min/1.73m^2^	0.05	0.622			− 0.19	0.077	− 0.10	0.061	0.23	0.030	0.21	0.052
LVM, g	0.39	0.001	**0.22**	**0.032**	0.29	0.015	**0.28**	**0.007**	− 0.25	0.034	**− 0.22**	**0.002**

β is adjusted regression coefficient. Variables with bold font indicate statistical significance in multivariable analysis. ^#^. Changes in dependent variables per 10 units increase. ^&^. Subjects with body mass index ≥ 25 kg/m^2^ were classified as obese group that proposed by the World Health Organization for Asian populations. ^*^. NT-proBNP is log-transformed before being included in the regression analysis

Abbreviations as listed in Table 3

## Discussion

The present study highlights evidence supporting sex-related differences in LV geometry and contractility in patients with HFpEF comorbid with DM. The main findings were as follows: (1) Despite having similar LVEF, women displayed an increased LVM index and a trend toward more concentric remodeling/hypertrophy in the context of DM than men. (2) LV contractility in women was more likely to be affected by DM than that in men, with worse systolic strain noted in women with DM than in men with DM. Furthermore, the presence of DM deteriorated LV contractility in women. In contrast, no significant differences in LV contractility were observed between men with DM and men without DM. (3) After adjustment for several factors, DM remained the main independent determinant of impaired myocardial contractility in women but not in men.

### Sex-related differences in LV remodeling in the context of DM

Previous studies have reported sex differences in cardiac structure and function, as demonstrated by lower LV volumes and LVM, with more diastolic dysfunction in women with HFpEF [7, 8]. The present study found similar changes in cardiac structure and function in the non-DM subgroup. To date, the underlying mechanisms for sex differences in cardiac remodeling in HFpEF are not well understood. However, since most patients with HFpEF are aged > 60 years, some evidence suggests that hormonal differences and biohormonal system activity may play a key role in cardiac remodeling and predominantly affect women’s hearts [17–19]. Moreover, comorbid cardiovascular risk factors, such as DM, represent other major factors responsible for LV remodeling in HFpEF development [20]. In recent studies comparing HFpEF patients with and without DM, those with DM seem to present with increased LVM [21–23]. Nevertheless, the sex differences in LV remodeling in the context of DM are unclear. The present study provided further evidence to support the susceptibility of women to develop LVH and concentric remodeling/hypertrophy in response to DM.

### Sex-related differences in the effect of DM on LV contractile function

In terms of HFpEF, strain analysis allows the detection of systolic dysfunction in the absence of an overt reduction in LVEF and shows prognostic value in predicting adverse outcomes [24, 25]. However, available data regarding the sex-related impact of coexisting DM on LV contractility in patients with HFpEF remain limited. To the best of our knowledge, the present study is the first to perform separate analyses of myocardial contractile differences and explanatory determinants for impaired myocardial contractility between sexes. We observed that the magnitude of global systolic strain was similar between sexes in the non-DM subgroup, which was in line with the findings of a previous study by Gori et al. after adjusting for the presence of DM [6]. Interestingly, when considering the DM status in the overall cohort, women with DM exhibited worse systolic strain than men with DM. Furthermore, we also found that the coexistence of DM aggravates LV contractility in women with HFpEF and DM compared to women with HFpEF without DM.

The underlying mechanisms of the adverse effect of DM on LV systolic function are not fully understood. HFpEF comorbid with DM could induce a broad context of metabolic disorder, oxidative stress, and a systemic inflammatory state, resulting in excitation-contraction coupling impairment, extracellular matrix fibrosis, and microvasculature dysfunction, thereby promoting myocardial contractility impairment [3, 23, 26]. Based on the observation in our study that DM has a greater adverse effect on LV contractility in women than in men, the decline in the estrogen level at menopause together with the presence of DM may be responsible for this difference [27]. During this period, metabolic syndrome and chronic activation of the renin-angiotensin-aldosterone system may play a key role in the exacerbation of LV contractility [28].

### Sex-related differences in determinants of impaired LV contractile function

The association between age and reduced systolic strain in both women and men could be explained by age-dependent myocyte loss and extracellular matrix remodeling with interstitial fibrosis [17]. However, a previous study by Roy et al. reported that disease-related diffused myocardial fibrosis is another pathological characteristic of LVH [29], which could in turn support our finding that the degree of LVH remains associated with reduced systolic strain in both sexes after adjusting for age. Women had higher median NT-proBNP levels than men in the present study, despite the similar duration of HF. This finding may be explained by the higher prevalence of DM in women. The independent association between NT-proBNP levels and reduced systolic strain could reveal high LV end-diastolic wall stress in both sexes [15].

In our study, it was particularly notable to identify the independent determinant of DM for impaired LV systolic strain in women but not in men. This finding may reflect the susceptibility to LV systolic dysfunction in women in the context of DM. Supporting evidence reveals that coronary microvasculature dysfunction is more frequently observed in women [30]. Therefore, it is conceivable that, in the context of DM, superimposed effects could significantly aggravate LV contractility in women with DM. This finding highlights the importance of sex-specific stratification and treatment strategies [31]. In addition, the lack of association between hypertension and systolic strain may be related to the fact that the majority of patients with hypertension were in good control of their blood pressure.

In men, obesity remains an independent variable related to impaired LV systolic strain. Obesity has been increasingly recognized as a key factor contributing to the evolution and progression of HFpEF [32]. Unlike the data from Western patient populations from registries or community-based studies [7, 8, 33, 34], our study reported higher BMI in men than in women, which suggests that the prevalence of obesity varies by race. The effect of sex on the obese phenotype of HFpEF needs further investigation. Moreover, a previous large-sample study reported that CAD is more common in men, which is in line with the findings of the current study [35]. Our current analysis showed that CAD was independently associated with impaired GLS but not with GCS or GRS. This finding highlights the prognostic value of GLS in predicting cardiovascular events [25]. The association between CAD and impaired GLS in our study could provide evidence on why men appear more likely than women to have sudden cardiac death related to CAD [7].

### Implications for DM treatment in women with HFpEF

Our study emphasizes the fundamental differences between sexes driven by cardiovascular risk factors, and HFpEF comorbid with DM in women is a high-risk phenotype of cardiac failure that may require specific treatment. Regardless, after medical management, women show better LVEF improvement than men in nearly all trajectories, with a more pronounced inverted U-shaped pattern [36]. Moreover, following a healthy lifestyle pattern is associated with lower risks of HFpEF among postmenopausal women [37]. Therefore, early intervention with lower thresholds may provide potential benefits.

## Limitations

Our study has several limitations. First, this was a single-center study performed with a relatively small number of patients. The findings should be validated by future studies with a large study sample. Second, our study indicates that DM has a greater adverse effect on LV systolic strain in women than in men, and longitudinal studies will be needed to investigate how these differences in systolic strain impairment affect long-term outcomes in women and men with DM. Third, we did not perform echocardiographic strain analysis. It would be interesting to compare the myocardial strain data between these two modalities (echocardiography vs. cardiac MRI) in the future. Finally, since echocardiographic data were obtained from the medical records of our hospital, an analysis of intra- and interobserver variability of echocardiographic measurements was not carried out in this study, which may have implications on the echocardiographic results. Moreover, some items, such as invasive hemodynamic analyses, that may be helpful in risk stratification were not included in this study.

## Conclusions

In conclusion, this study highlights the sex-related differences in LV systolic function in patients with HFpEF comorbid with DM, with DM exerting a greater adverse effect on LV contractility in women than in men. The current study indicates that lower thresholds for initiating treatment may be required in the high-risk patient population of women with DM.

## Data Availability

The datasets used and analyzed during the current study are available from the corresponding author on reasonable request.

## Abbreviations

HF: Heart failure
HFpEF: Heart failure with preserved ejection fraction
LV: Left ventricular
DM: Diabetes mellitus
NT-proBNP: Pplasma amino-terminal pro-B-type natriuretic peptide
LVEF: Left ventricular ejection fraction
E: peak early diastolic filling velocity
e’: Peak early diastolic mitral annular velocity
LA: Left atrial
LVH: Left ventricular hypertrophy
LVEDV: Left ventricular end-diastolic volume
LVESV: Left ventricular end-systolic volume
LVSV: Left ventricular stroke volume
LVM: left ventricular mass
GLS: Global longitudinal strain
GCS: Global circumferential strain
GRS: Global radial strain
LAV: Left atrial volume
A: Late diastolic filling velocity during atrial contraction
PASP: Pulmonary artery systolic pressure
RWT: Relative wall thickness
IQR: Interquartile range
BMI: Body mass index
CAD: Coronary artery disease

## References

[1] Shah KS, Xu H, Matsouaka RA (2017). Heart failure with preserved, borderline, and reduced ejection fraction: 5-year outcomes. J Am Coll Cardiol.

[2] Parikh KS, Sharma K, Fiuzat M (2018). Heart failure with preserved ejection fraction expert panel report: current controversies and implications for clinical trials. JACC Heart Fail.

[3] McHugh K, DeVore AD, Wu J, Matsouaka RA, Fonarow GC, Heidenreich PA, Yancy CW, Green JB, Altman N, Hernandez AF (2019). Heart failure with preserved ejection fraction and diabetes: JACC state-of-the-art review. J Am Coll Cardiol.

[4] Nouraei H, Rabkin SW (2021). A new approach to the clinical subclassification of heart failure with preserved ejection fraction. Int J Cardiol.

[5] Tibrewala A, Yancy CW (2019). Heart failure with preserved ejection fraction in women. Heart Fail Clin.

[6] Gori M, Lam CS, Gupta DK (2014). Sex-specific cardiovascular structure and function in heart failure with preserved ejection fraction. Eur J Heart Fail.

[7] Dewan P, Rørth R, Raparelli V (2019). Sex-related differences in heart failure with preserved ejection fraction. Circ Heart Fail.

[8] Beale AL, Nanayakkara S, Kaye DM (2019). Impact of sex on ventricular-vascular stiffness and long-term outcomes in heart failure with preserved ejection fraction: TOPCAT Trial substudy. J Am Heart Assoc.

[9] Tadic M, Cuspidi C, Plein S, Belyavskiy E, Heinzel F, Galderisi M (2019). Sex and heart failure with preserved ejection fraction: from pathophysiology to clinical studies. J Clin Med.

[10] Pepine CJ, Merz CNB, El Hajj S (2020). Heart failure with preserved ejection fraction: similarities and differences between women and men. Int J Cardiol.

[11] Cifkova R, Pitha J, Krajcoviechova A, Kralikova E (2019). Is the impact of conventional risk factors the same in men and women? Plea for a more gender-specific approach. Int J Cardiol.

[12] Bank IEM, Gijsberts CM, Teng TK (2017). Prevalence and clinical significance of diabetes in Asian versus white patients with heart failure. JACC Heart Fail.

[13] Lindman BR, Dávila-Román VG, Mann DL (2014). Cardiovascular phenotype in HFpEF patients with or without diabetes: a RELAX trial ancillary study. J Am Coll Cardiol.

[14] Lorenzo-Almorós A, Tuñón J, Orejas M, Cortés M, Egido J, Lorenzo Ó (2017). Diagnostic approaches for diabetic cardiomyopathy. Cardiovasc Diabetol.

[15] Pieske B, Tschöpe C, de Boer RA (2019). How to diagnose heart failure with preserved ejection fraction: the HFA-PEFF diagnostic algorithm: a consensus recommendation from the Heart Failure Association (HFA) of the European Society of Cardiology (ESC). Eur Heart J.

[16] Schulz-Menger J, Bluemke DA, Bremerich J (2020). Standardized image interpretation and post-processing in cardiovascular magnetic resonance—2020 update: Society for Cardiovascular Magnetic Resonance (SCMR): Board of trustees task force on standardized post-processing. J Cardiovasc Magn Reson.

[17] Keller KM, Howlett SE (2016). Sex differences in the biology and pathology of the aging heart. Can J Cardiol.

[18] Zhao D, Guallar E, Ouyang P (2018). Endogenous sex hormones and incident cardiovascular disease in post-menopausal women. J Am Coll Cardiol.

[19] Gregori M, Tocci G, Marra A, Pignatelli G, Santolamazza C, Befani A, Ciavarella GM, Ferrucci A, Paneni F (2013). Inadequate RAAS suppression is associated with excessive left ventricular mass and systo-diastolic dysfunction. Clin Res Cardiol.

[20] Tromp J, Teng TH, Tay WT (2019). Heart failure with preserved ejection fraction in Asia. Eur J Heart Fail.

[21] Yap J, Tay WT, Teng TK (2019). Association of diabetes mellitus on cardiac remodeling, quality of life, and clinical outcomes in heart failure with reduced and preserved ejection fraction. J Am Heart Assoc.

[22] Shen L, Rørth R, Cosmi D (2019). Insulin treatment and clinical outcomes in patients with diabetes and heart failure with preserved ejection fraction. Eur J Heart Fail.

[23] Lejeune S, Roy C, Slimani A (2021). Diabetic phenotype and prognosis of patients with heart failure and preserved ejection fraction in a real-life cohort. Cardiovasc Diabetol.

[24] Shah AM, Claggett B, Sweitzer NK (2015). Prognostic importance of impaired systolic function in heart failure with preserved ejection fraction and the impact of spironolactone. Circulation.

[25] Kammerlander AA, Donà C, Nitsche C (2020). Feature tracking of global longitudinal strain by using cardiovascular MRI improves risk stratification in heart failure with preserved ejection fraction. Radiology.

[26] Li XM, Jiang L, Guo YK (2020). The additive effects of type 2 diabetes mellitus on left ventricular deformation and myocardial perfusion in essential hypertension: a 3.0 T cardiac magnetic resonance study. Cardiovasc Diabetol.

[27] de Kat AC, Dam V, Onland-Moret NC, Eijkemans MJ, Broekmans FJ, van der Schouw YT (2017). Unraveling the associations of age and menopause with cardiovascular risk factors in a large population-based study. BMC Med.

[28] Sabbatini AR, Kararigas G (2020). Menopause-related estrogen decrease and the pathogenesis of HFpEF: JACC review topic of the week. J Am Coll Cardiol.

[29] Roy C, Slimani A, de Meester C (2018). Associations and prognostic significance of diffuse myocardial fibrosis by cardiovascular magnetic resonance in heart failure with preserved ejection fraction. J Cardiovasc Magn Reson.

[30] Zijlstra LE, Bootsma M, Jukema JW, Schalij MJ, Vliegen HW, Bruschke AVG (2019). Chest pain in the absence of obstructive coronary artery disease: a critical review of current concepts focusing on sex specificity, microcirculatory function, and clinical implications. Int J Cardiol.

[31] Sara JD, Taher R, Kolluri N, Vella A, Lerman LO, Lerman A (2019). Coronary microvascular dysfunction is associated with poor glycemic control amongst female diabetics with chest pain and non-obstructive coronary artery disease. Cardiovasc Diabetol.

[32] Packer M (2018). Leptin-aldosterone-neprilysin axis: identification of its distinctive role in the pathogenesis of the three phenotypes of heart failure in people with obesity. Circulation.

[33] Pandey A, Omar W, Ayers C (2018). Sex and race differences in lifetime risk of heart failure with preserved ejection fraction and heart failure with reduced ejection fraction. Circulation.

[34] Merrill M, Sweitzer NK, Lindenfeld J, Kao DP (2019). Sex differences in outcomes and responses to spironolactone in heart failure with preserved ejection fraction: a secondary analysis of TOPCAT trial. JACC Heart Fail.

[35] Goyal P, Paul T, Almarzooq ZI (2017). Sex- and race-related differences in characteristics and outcomes of hospitalizations for heart failure with preserved ejection fraction. J Am Heart Assoc.

[36] Julián MT, Alonso N, Lupón J (2020). Long-term LVEF trajectories in patients with type 2 diabetes and heart failure: diabetic cardiomyopathy may underlie functional decline. Cardiovasc Diabetol.

[37] Noel CA, LaMonte MJ, Roberts MB (2020). Healthy lifestyle and risk of incident heart failure with preserved and reduced ejection fraction among post-menopausal women: the Women’s Health Initiative study. Prev Med.

